# Two sides of the coin: patient and provider perceptions of health care delivery to patients from culturally and linguistically diverse backgrounds

**DOI:** 10.1186/1472-6963-12-322

**Published:** 2012-09-18

**Authors:** Nera Komaric, Suzanne Bedford, Mieke L van Driel

**Affiliations:** 1 Faculty of Health Sciences and Medicine, Bond University, Gold Coast, QLD, Australia; 2 Discipline of General Practice, School of Medicine, The University of Queensland, Brisbane, Australia; 3 Department of General Practice and Primary Health Care, Ghent University, Ghent, Belgium

## Abstract

**Background:**

Australia is a culturally diverse nation with one in seven Australians born in a non-English speaking country. Culturally and Linguistically Diverse (CALD) populations are at a high risk of developing preventable chronic diseases such as cardiovascular disease, type 2 diabetes mellitus, renal disease, and chronic respiratory disease, especially communities from the Pacific Islands, the Middle East, North Africa, the Indian subcontinent and China. Previous studies have shown that access to services may be a contributing factor. This study explores the experiences, attitudes and opinions of immigrants from different cultural and linguistic backgrounds and their health care providers with regard to chronic disease care.

**Methods:**

Five focus groups were conducted comprising participants from an Arabic speaking background, or born in Sudan, China, Vietnam or Tonga. A total of 50 members participated. All focus groups were conducted in the participants’ language and facilitated by a trained multicultural health worker. In addition, 14 health care providers were interviewed by telephone. Interviews were digitally recorded and transcribed. All qualitative data were analysed with the assistance of QSR NVivo 8 software.

**Results:**

Participants were generally positive about the quality and accessibility of health services, but the costs of health care and waiting times to receive treatment presented significant barriers. They expressed a need for greater access to interpreters and culturally appropriate communication and education. They mentioned experiencing racism and discriminatory practices. Health professionals recommended recruiting health workers from CALD communities to assist them to adequately elicit and address the needs of patients from CALD backgrounds.

**Conclusions:**

CALD patients, carers and community members as well as health professionals all highlighted the need for establishing culturally tailored programs for chronic disease prevention and management in CALD populations. Better health care can be achieved by ensuring that further investment in culturally specific programs and workforce development is in line with the number of CALD communities and their needs.

## Background

Chronic disease is a major and growing health problem. According to the World Health Organisation, 35 million deaths due to major chronic disease were reported globally in 2005, with the highest toll evident in low and middle income countries [1]. In Australia, the aging population and changes in lifestyle that have emerged over the past decades call for a focus on chronic diseases in planning and delivery of health care services. Several Australian states have responded with strategic plans to tackle the challenges posed to the health care system. Queensland’s Chronic Disease Strategy 2005–2015 mentions six different target groups, including people from culturally and linguistically diverse backgrounds (CALD) [2]. In the Australian context, individuals from a CALD background identify as having specific cultural or linguistic affiliation by virtue of their place of birth, ancestry, ethnic origin, religion, preferred language, or language spoken at home, or because of their parents’ identification on a similar basis.

Migrants to Australia undergo rigorous health checks before migration. Consequently, upon arrival in Australia, their health status is better than the Australian-born population, which is known as the 'healthy migrant effect' [3] A recent Australian study, however, shows that the longer immigrants spend in Australia, the closer their health approximates that of the Australian-born population [4]. CALD populations often experience a higher burden of disease at the system level, such as a lack of access to services and lack of appropriate information to make informed decisions, which impacts significantly on their health and quality of life [5].

Delivering effective health services to members of the CALD community is complex because cultural and language barriers impede the identification, targeting and delivery of services. Clients from non-English-speaking backgrounds are disadvantaged in terms of access and quality of services compared to the mainstream English speaking population [6] this is expressed in lower health levels. In addition, acculturation may be an important risk factor in chronic disease morbidity for immigrants from non-English speaking backgrounds.

As Australia becomes more culturally and linguistically diverse society, the health care system needs to be responsive to CALD patients’ health needs. Failure to provide culturally competent services would lead to major health care disparities between CALD and mainstream populations. Although there is no single definition of cultural competency, in broader terms it describes the capacity to provide professional and adequate services to populations with diverse values, beliefs, behaviours, cultural identity and ethnic identity [7]. Ideally provision of such services should be embedded throughout the health care system to the extent that cultural competency is apparent from the individual provider-patient interface through to policy development at the governmental level.

We believe there are two key barriers to the provision of adequate health care to CALD populations. The first is at the level of the health care system itself where there is a lack of cultural diversity in the health care workforce. This manifests in an under-representation of ethnic minorities throughout all levels of the healthcare system with only recent leadership being offered to address the lack of cultural competency at governmental levels [8].

The second significant barrier lies within the CALD communities. According to Nutbeam [9], there is a clear correlation between a person’s health literacy and health status. In brief, health literacy is defined as the set of literacy and numeracy skills that enable a person to comprehend their health status and be proactive addressing their health care needs [10]. Whilst low levels of health literacy are apparent throughout the population in Australia, the level of health literacy of persons whose first language is not English is significantly poor. For instance, only 3.4% of 65–74 year olds whose first language is not English could be regarded as being health literate compared to 17.4% in the same age group in the mainstream population [11]. This comparatively low level of health literacy creates a further impediment to the provision of adequate care for members of the CALD community and has considerable implications for the capacity of these patients to self-manage chronic disease states in particular.

In this study we aimed to describe the challenges people from CALD communities face regarding treating and preventing a chronic disease and what barriers they experience and perceive with regard to access to health services. To distinguish this study from others we sought to provide a more comprehensive picture of the challenges faced when providing health care to this vulnerable population. To this end, we investigated the perceptions not only of members of the CALD communities but also those of the health professionals involved in their care.

## Methods

We conducted focus group discussions with people from diverse linguistic and cultural backgrounds and interviews with health care providers who service CALD populations.

### Focus groups

#### Selection criteria

Participants were eligible if they belonged to a CALD community, identified by country of birth; language other than English spoken at home and English language proficiency, and lived in a region with a high prevalence of chronic disease [12]. All participants were registered in the database of the Ethnic Communities Council of Queensland (ECCQ) and either suffered from a chronic disease, cared for a patient with chronic disease or had an interest in chronic disease service delivery. The chronic diseases of interest were limited to seven lifestyle preventable diseases namely type 2 diabetes, heart failure, stroke, coronary heart disease, renal failure, chronic obstructive pulmonary disease and asthma which have been identified as priorities in the Queensland Chronic Disease Strategy 2005–2015 [2].

#### Sample population

A total of 50 participants took part in five ethnically homogenous focus groups each comprising 8–11 participants. In collaboration with the community organisations active in the health promotion for CALD communities, we selected a convenience sample of Arabic speaking, Chinese, Sudanese, Tongan and Vietnamese groups. These ethnic groups were targeted based on the high prevalence of chronic disease within these populations in south-east Queensland.

#### Data collection

Participants were fully apprised of the research aims and protocol before providing written, informed consent in accordance with the NHMRC Statement on Human Experimentation [13].

The focus groups were conducted in the community languages and followed a semi-structured format with the facilitator posing open-ended questions categorised into the major themes under consideration in the study: chronic disease awareness, health care system literacy, utilisation of the health care system, awareness of types of health care provider, preferred type of provider, access issues, experience of the health care system and suggestions for improvement. This set of open-ended questions was derived from a previously conducted quantitative study as a consequence of a numerous consultations with CALD communities in Queensland.

Focus group sessions lasted approximately 90 min, were recorded, transcribed *verbatim* and translated into English. A bilingual person from each community validated translated transcripts. The facilitators were recruited from the pool of Multicultural Health Workers (MHW) employed by the ECCQ CALD Chronic Disease program to work with the specific CALD communities. Each MHW arranged a note taker from the community who was literate in the community’s language. The decision to run focus groups to collect data from the CALD communities was based on similar qualitative studies which have found that this approach provides a supportive environment in which community members are comfortable to freely express their views [14,15].

### Health professional interviews

#### Selection criteria

Health professionals were selected from the health districts of the focus group participants' residence. Fifteen professionals agreed to be interviewed with one later withdrawing from the study.

#### Interview methods

Participants were informed of the research aims and protocols before giving verbal consent. Interviews were conducted and recorded in English via telephone by an independent consultant using a structured interview schedule. Questions centred on their professional context, experience with patients from CALD communities, attitudes of their organisation towards CALD communities, perception of disease in CALD communities, factors impacting treatment and suggestions for improving health care provision to CALD communities. Each interview took approximately thirty minutes and was transcribed *verbatim* by the interviewer.

#### Data analysis and reporting

Focus group and interview data were analysed and themes identified separately with the assistance of qualitative analysis software QSR NVivo 8 (QSR International PTY Ltd, Melbourne, Australia). Thematic analysis was used to identify emerging themes in focus group and interview data. Transcripts of both the interview and focus group data were read several times with issues raised by both providers and focus group participants coded to themes. This was an interactive process with thematic categories added or refined with each consideration of the transcripts. These themes were identified independently by two investigators and any discrepancies in analysis were discussed amongst the investigators as the analysis progressed until a resolution regarding thematic categorisation was achieved. This process was repeated until no further themes could be identified by either investigator, suggesting thematic saturation had been achieved in this sample. The transparency of the analysis is indicated by extensive use of verbatim quotes in the text.

#### Ethics

Ethics approval for the study was obtained from the Bond University Human Research Ethics Committee.

## Results

### Study population

#### Focus groups

The age of participants ranged from 17 to 79 years old. Participants had immigrated to Australia between 1 and 38 years ago, had no to high standards of English proficiency and were from a broad range of educational backgrounds.

The proportion of participants reporting a current chronic disease ranged from 3 out of 9 participants (Chinese) to 10 out of 11 participants (Sudanese). Members within each group reported suffering from a variety of chronic conditions or had family members with chronic disease (Table 1), with hypertension, hypercholesterolemia and type 2 diabetes mellitus being well represented.

**Table 1:** General demographic characteristics of focus group participants as established by selection criteria

**Focus Group**	**No. in group**	**Year of birth (range)**	**Years since arrival (median, range)**	**Spoken English proficiency**	**Highest education achievement**	**No. with disease**
Arabic	8	1954-1992	12 years, 1–37 years	All good or better	All high school or higher	4
Sudanese	11	1948-1977	6 years, 3–14 years	4 poor or worse	5 high school or lower	10
Chinese	9	1930-1983	5 years, 2–17 years	2 poor or worse	All high school of lower	3
Vietnamese	12	1930-1969	14 years, 9–30 years	5 poor or worse	8 high school or lower	6
Tongan	10	1953-1977	19.5 years, 3–38 years	3 poor or worse	7 high school or lower	4

#### Professional context of health providers

Twelve of the 14 health care providers interviewed were female, half were Australian and all worked in a variety of settings including community health and tertiary institutions (see Table 2). Exposure to CALD patients ranged from very limited to comprising the majority of clientele. The level of focus their workplaces had on treating CALD communities appeared to correspond to the proportion of CALD patients in their practice.

**Table 2:** Health care provider profiles

**Code**	**Gender**	**Occupation**	**Institution**	**Ethnic background**	**Exposure to CALD communities**
HP01	female	Nutritionist	Population Health	Not CALD	Indirectly through project work
HP02	female	Anaesthetist, pre-admission	Public hospital	English	Directly, 20-30%
HP03	female	Nutritionist	Community & Primary Health services	Australian	Indirectly through consultation for resource development
HP04	male	GP	Community general practice	Australian (bilingual, Spanish)	Directly, 100%
HP05	female	GP	General Practice	Australian	Directly, <10%
HP06	female	Nutritionist	Population Health	Dutch	Indirectly, previous direct experience
HP07	female	Nurse (surgery)	Private hospital	Former Yugoslav	Directly, limited (<1%)
HP08	female	Audiologist	Public hospital	Australian	Directly,30-40%
HP09	female	Lifestyle management	Community Health	Caucasian	Directly,10-15%
HP10	female	GP	Private practice & refugee clinic	Australian (bilingual, Spanish)	Directly, 100% in refugee clinic, minority in general practice
HP11	male	Radiographer	Public hospital	Centro-eastern European	Directly, <10% patients
HP12	female	Occupational therapist	Community Health	Not CALD	Directly, 30-100% depending on project
HP13	female	Cardiac scientist	Public Hospital	Macedonian	Directly,15-20%
HP14	female	Coordinator, lifestyle programs	Community Health	Australian	Directly, 5-10%

Workplace attitudes towards CALD patients were viewed as positive with the “*attitude …getting much better.*” (HP13) This appeared to be particularly the case in organisations that have a greater exposure to CALD patients. In contrast, it was noted by one GP that:

"“In general practice we are all individual…. I think our organisation deals better with people from our own culture than with people from CALD groups. It’s not on purpose, it’s just that they are a minority.” (HP10)"

### Perception of disease in CALD communities

#### Chronic disease

All focus groups were aware of a variety of chronic and acute diseases within their communities, in particular type 2 diabetes and hypertension (see Table 3). In general, all providers who dealt with CALD patients, treated CALD patients with chronic disease. Whilst there was a perception amongst providers that the prevalence of chronic disease was lower in CALD compared to mainstream patient populations, prevalence appeared to depend on the ethnicity. For example, in some settings, disease presentation appeared to depend on patient’s region of origin with *“CALD clients …from Europe.. hav(ing) the same diseases that we have”* (HP10) compared with, for example, *“the Burmese … they don’t have those problems.”*(HP10) The effects of acculturation were apparent in the belief that the”…*chronic disease profile that is typical in the mainstream population will be adopted by the newly arrived migrants and CALD communities*” (HP3) because a *“lot of new arrivals in Australia adopt the lifestyle of (the) host country.”* (HP6)

**Table 3:** Disease presence, perception of disease or related symptom(s) prevalence in focus group communities and health service familiarity

**Focus Group**	**Disease presence in focus group participants**	**Disease/symptom prevalence within ethnic community**	**Disease/symptom prevalence within youth of ethnic community**	**Health service familiarity**
Arabic	In-Scope: Hypertension, Hypercholesterolaemia, Type 2 Diabetes Other: Arthritis, Asthma, Cancer	In-Scope & lifestyle: Type 2 Diabetes, Hypertension, Hypercholesterolaeia Other: Rheumatic heart disease, Cancer Mental Health: Depression, Stress	In-Scope: Other: AIDS, Asthma	Primary: Medical centres, GPs Specialist: general awareness Emergency & Tertiary: Hospitals Diagnostic: Pathology, Radiology Allied & Other Health: Pharmacy Community & NGO: Diabetes Centre, MCHW
Sudanese	In-Scope: Hypertension, Hypercholesterolaemia, Type 2 Diabetes Other: Asthma, Cancer, Stroke, unspecified liver disease	In-Scope & lifestyle: Type 2 Diabetes, Hypertension, Hypercholesterolaemia Other: Asthma, Cancer, Infectious Diseases *eg Hep B,* Parasitic Diseases *eg Malaria,* Malnutrition, unspecified abdominal & back pain Mental Health: Depression, Chronic grief, Stress	In-Scope & lifestyle: Obesity Other: Asthma, Epilepsy, Infectious Diseases eg *Hep B,* Parasitic Diseases eg *Malaria,* Malnutrition Mental Health: Depression, Chronic grief, Stress	Primary: Medical centres, GPs Specialist: general awareness Emergency & Tertiary: Ambulance, Hospitals Allied & Other Health: Occupational Therapy, Dentistry, Mid-wifery Community & NGO: Community Health Centre, Arthritis, Asthma, Kidney & Heart Foundations, MCHW
Chinese	In-Scope: Hypertension Other: Arthritis	In-Scope & lifestyle: unspecified chronic disease, Type 2 Diabetes, undefined kidney disease, Pulmonary emphysema, Overweight Other: Arthritis	In-Scope & lifestyle: Other: only undefined congenital conditions & arthritis mentioned	Primary: Medical centres, GPs Specialist: Gynaecology Emergency & Tertiary: Hospitals Diagnostic: Pathology, Radiology Allied & Other Health: Dentistry, Optometry, Pharmacy Mental Health: Psychology
Vietnamese	In-Scope: Hypertension, Hypercholesterolaemia, Type 2 Diabetes Other: Arthritis, Stroke, Hepatitis B	In-Scope & lifestyle: unspecified chronic disease Other: Joint-related disorders, dementia Mental Health: Depression, Stress	In-Scope & lifestyle: Smoking & alcohol related diseases Other: Chronic back pain	Primary: GPs Specialist: general awareness Emergency & Tertiary: Hospitals Diagnostic: Pathology, Radiology Allied & Other Health: Physiotherapy, Podiatry, Dentistry, Optometry, Midwifery, Occupational Therapy, Acupuncture Community & NGO: Home nurse, Hot line health service, Community Health Centre, MCHW
Tongan	None cited	In-scope & lifestyle: Overweight Hypertension, Type 2 Diabetes, Hypercholesterolaemia, Smoking & alcohol related disease Other: Cancer, Dementia Mental health: not different from broader community	In-scope & lifestyle: Overweight, alcohol related problems Other: Asthma, Eczema	Primary: Medical centre, GPs Emergency & Tertiary: Ambulance, Hospitals Allied: Physiotherapy Community & NGO: Community Health Centre Mental Health: Counselling, Psychiatry

#### Chronic disease and the younger members of CALD communities

Asthma was regarded more as a problem in younger members of the Arabic, Sudanese and Tongan communities (Table 3). The importance of infectious and parasitic disease was raised by the Sudanese community along with malnutrition. Vietnamese and Tongan communities perceived their youth as being at risk of acquiring lifestyle related problems. There was no particular mention of disease prevalence in younger members of the CALD communities in the health-provider interviews.

#### Perception of mental health problems in CALD communities

Arabic and Sudanese participants emphasized the importance of mental health. Both communities identified war in their region of origin as one of the causes of “depression, stress and anger.” (Arabic)

"“The war has affected us and many of us are traumatised and have gone through agony/suffering and torture, we came here with high hope of good opportunities unfortunately it did not work out as we expected due to cultural differences”. (Sudanese)"

The Sudanese focus group mentioned loneliness, dislocation and isolation from relatives in Sudan as reasons for chronic grief, stress and depression. They perceived their youth as being particularly vulnerable to mental health problems citing the high level of frustration with the challenges presented at school as a key contributing factor.

Concerns about mental health were also raised by health-providers who treated traumatised CALD patients. There was a perception that previous trauma experienced by refugees who *“have been victimised or tortured in their own country”* (HP14) had a negative impact on assimilation. This, in addition to the lack of appropriate interpreting services, was believed to impact on their capacity to provide adequate treatment to these patients:

"“They have a lot of traumatisation symptoms and it is a difficult task to ask them about the sort of sexual abuse or abuse when they were younger or their background, and then I am limited with the phone interpreter who’s often male asking them big questions.” (HP5)"

Providers indicated they needed support either through appropriate training or knowing where to refer traumatised patients.

### Familiarity with health services

All focus group participants were familiar with a variety of health services (Table 3). These included providers such as GPs, dental clinics and hospitals as well as some allied health services. Mental health services were only mentioned by the Chinese and Tongan participants. Only Vietnamese, Arabic and Sudanese participants consulted multicultural health workers (MHW) or clinicians. In general, with the exception of the Arabic and Sudanese community, knowledge of community based Non-Governmental Organisations (NGOs) providing health care services was poor. Despite a generalised knowledge of health services, some focus groups (Sudanese, Tongan and Chinese) reported a lack of understanding of how the health care system in Australia worked. Furthermore, a low level of health care system literacy was cited by some health care providers as having a detrimental impact on health care for members of the CALD communities.

### Accessibility to health care services

Accessibility to health care services were analysed in terms of proximity, availability of services and affordability. These themes are summarised in Table 4.

**Table 4:** Summary of perceptions of the accessibility of health care services by focus group participants and providers

**EMERGENT THEMES**	**Arabic**	**Sudanese**	**Tongan**	**Chinese**	**Vietnamese**	**Health-care Providers**
Proximity of health services						
Convenient	x	x		x	x	
Far away		x				x
Will travel to preferred provider		x	x			x
Availability of Services						
Lack of available providers overall	x	x		x	x	x
Long waiting list for specialists	x			x	x	x
Long waiting time for treatment in emergency departments	x	x	x	x	x	x
Long operation waiting lists	x	x			x	
Shortage of beds in hospitals	x	x	x			
Lack of knowledge of health system/services		x	x	x		x
Socioeconomic factors impacting on access to healthcare						
Expense of services	x	x		x	x	x
Expense of medication	x	x				x
Restricted access to bulk-billing services						x

#### Proximity of health services

Limited access to appropriate health care services was cited as a significant problem for CALD populations in South-East Queensland. Whilst many focus group participants perceived health services easy to physically access, Sudanese participants found public transport services inadequate and taxi cabs too expensive. The difficulty in physically accessing health services was also raised by health care providers not only for CALD communities but also the mainstream population.

#### Availability of services

Focus group participants cited the inadequate supply of doctors and nurses as a primary causative factor underlying restricted access to services. This was reported as contributing to long waiting lists for specialist services, elective surgery and the time wasted waiting in the Emergency Department. Health providers mentioned this was not only impacting CALD communities:

"“a lot of people …not hav(ing) access to primary and secondary prevention of chronic disease.” (HP6)"

Restricted access to health care services for CALD patients was seen to be further compounded by the perceived reluctance of practices willing to treat CALD patients as it *“would affect their demographics and their payments from the Government and they don’t bulkbill.”* (HP5)

Access to adequate services was also seen to be limited by a lack of health care providers capable of servicing CALD communities, for example:

"“…in Brisbane, I think, there are four or five Spanish speaking doctors providing primary care.. I think they have difficulties with access”. (HP4)"

#### Socioeconomic factors affecting access

Expense of services or treatment was raised by a number of focus group participants, with medications, for example, being too expensive, regardless of socioeconomic circumstances. Specialist services (Sudanese), dental (Vietnamese) and private tertiary treatment (Chinese, Arabic) were also regarded as prohibitively expensive. Restricted access to bulk-billing services (where there is no out-of-pocket contribution from the patient) was also of concern to providers given the low socio-economic status of a significant proportion of the CALD community.

Impoverished circumstances were seen to not only impact access to appropriate health care but also to adequate nutrition, for example:

"“…a number of CALD people find it difficult to get a job, so financial income, financial problems, access to affordable and healthy food is also a problem…” (HP6)"

### Perceptions of patient-provider interactions

In general, focus group participants and providers were positive about their experiences. Nevertheless, all parties perceived linguistic and cultural incompatibilities had a detrimental impact on the provision of care. The extent to which these impacted on the perception of competency of the provider cannot be determined, however, concerns were raised regarding the professionalism of health care providers. These issues are discussed in further detail below and summarised in Table 5.

**Table 5:** Summary of perceptions of patient-provider interactions by focus group participants and providers

**EMERGENT THEMES**	**Arabic**	**Sudanese**	**Tongan**	**Chinese**	**Vietnamese**	**Health-care Providers**
Communication problems between patient and provider						
Language Barrier		x	x	x	x	x
Medical Terminology		x		x		x
Problems with interpreter				x		x
Not understanding accent of non-Western providers	x					
Lack of translated &/or health-related information	x	x	x	x	x	x
Cultural issues impacting on treatment						
General cultural misunderstanding on behalf of provider		x	x	x		x
Cultural differences decreasing compliance to treatment						x
Outright Discrimination		x			x	x
Feeling disrespected &/or undermined		x		x		
Discriminated against by non-Western providers				x	x	
General problematic patient-provider interactions						
Lack of professionalism on behalf of provider	x		x	x		x
Erroneous diagnoses		x	x	x		
Problems with health provider not operating to appointment schedule	x			x		
Patients not keeping appointments or often rescheduling						x
Lack of rapport		x		x	x	x
Inadequate provision of information of diagnosis/therapy by health provider		x	x	x	x	x
Inadequate treatment in hospitals		x			x	
Lack of training or referral services to enable delivery of appropriate care to trauma victims						x
Positive interactions between patient and provider						
Generally satisfied with treatment	x	x		x	x	x
Rapport established with provider	x			x		x
Rewarding experience						x
Adequate provision of information regarding diagnosis/therapy by health provider	x	x		x		x

#### Communication problems

Like access, the language barrier presented the main impediment to providing adequate health care to members of the CALD community. The extent to which linguistic incompatibilities impacted on the provision of health care varied within and across focus groups. Arabic participants did not report a problem with general communication to the same extent as the Sudanese, for example, but did cite difficulties in understanding the accents of English-speaking Indian or Chinese doctors.

When language presented a problem it affected all levels of treatment with communication breaking down between participants, support staff and health care providers:

"“Some receptionists are not friendly and are very rude especially when you cannot communicate properly due to language barrier of an African”. (Sudanese)"

The Tongan and Chinese communities also believed their elderly were particularly vulnerable to linguistic incompatibilities:

"“…people from Logan drive to Stafford to see Tongan doctor. It is the elderly who want to speak in Tongan. A language issue…” (Tongan)"

Language or communication barriers impacted on the patient’s capacity to convey health care needs even when the patient had a reasonable degree of English proficiency:

"“The doctor will ask you to describe what kinds of pains you have - I don’t know how to do that. So language is an obstacle for me, there are some professional words I don’t know” (Chinese)"

It was also perceived as having a detrimental effect not only on the patient’s understanding of the disease but also on compliance with treatment:

"“…people therefore don’t do the proper treatment because they don’t quite understand what it’s about due to the, just because of the language barrier.” (HP9)"

Overall, the provision of adequate interpreting services was seen by both focus group participants and health care providers as critical in improving delivery of services and increasing patients’ satisfaction with their care. Merely increasing interpreting services was recognised, however, as an overly simplistic solution to the complexities of delivering adequate health care to this patient population. Using interpreters presented its own difficulties:

"“We certainly have had conflict in the past with our qualified health professionals trying to assist and then the breakdown has been around the cultural worker...For example, I can say one sentence about chronic disease and then the health worker can spend fifteen minutes talking about the one sentence supposedly interpreting.” (HP14)"

Access to appropriate interpreting services was seen as especially critical when treating traumatised patients. Moreover, if interpreters who did not understand medical terminology were used, the consultation process was regarded as unsatisfactory. Chinese participants in particular felt it was important that interpreters had a good working knowledge of medical issues and terminology:

"“…at least the interpreters should have some medical background…” (Chinese)"

#### The impact of cultural incompatibility

The problems posed by cultural differences went beyond the language barriers, with cultural incompatibility being perceived as a major contributor to many of the misunderstandings between CALD patients and health care providers:

"“If there’s not cross understanding of the position of the patient, there can be miscommunication, misjudgement, and that can play a part as well….You don’t want your clinician to be making a judgement on something that’s actually a cultural issue rather than an attitude.” (HP8)"

Most focus group participants mentioned cultural differences between patient and provider as being problematic. Cultural incompatibilities between patient and provider were perceived by focus group participants to range from a simple lack of awareness on behalf of the GP: to racism in the primary care context:

"“…lack of cultural awareness for doctors, they don’t understand how I feel.” (Sudanese) to not sharing cultural norms with mainstream culture:"

"“I think there maybe also a cultural difference, for Australian, people who provide the service they still have their own right you know don’t like the doctor or something like that, but in Asian cultures they can’t do that.” (Chinese), to expressing the need for the health professionals with same cultural background:"

"“Involve experiences health practitioners from the Sudanese community who have knowledge on tropical diseases or common diseases in Sudan and addition understands the culture.” (Sudanese)"

"“Big problem-we are black and doctor white”(Tongan) or at the institutional level with support staff and providers being perceived as “bureaucratic and discriminating.” (Vietnamese)."

Chinese and Vietnamese focus group participants felt discriminated against by Asian and Australian providers. When faced with discrimination, participants felt undermined, disrespected and intimidated and felt that it had a negative influence on the perception of their care and the provider. Similar to language difficulties, cultural differences also impacted on compliance with treatment. For example, cultural reasons were seen to underpin non-attendance at appointments and non-compliance with treatment programs with *“some people depending on their cultural differences (being) reluctant to take on doctor’s advice.” (HP11)*

Failure to follow medication regimens was seen as a cultural issue, as well as patient preference for alternative methods of treatment. Concern was raised that *“they don’t often manage their chronic disease well because they’re trying to use alternative methods that don’t work in this culture where they now live*.” *(HP9)*

#### Perception of lack of professionalism

Whilst members of all groups reported feeling generally satisfied with their medical treatment and/or health services, many focus group participants believed that a lack of professionalism on behalf of providers detracted from or decreased the quality of their care. These problems were widespread and encompassed primary and tertiary health care settings and manifested mainly as diagnostic incompetence:

"“My father had been seeing his GP for long time but no improvement in his health, one of the community members advised us to see another GP or a specialist. I took my father to a specialist and he was diagnosed …with cancer.” (Sudanese);"

"“Three GPs diagnosed three different things” (Tongan);"

The inability or reluctance of health professionals to impart information regarding their diagnoses and treatments was stressed:

"“.if you didn’t ask them you cannot get the answer. For some people who don’t go to the doctor usually, they don’t know how to ask these questions. In my view, the doctor should tell you how to prevent directly.” (Chinese)"

Whether this perceived lack of professionalism was a consequence of linguistic and/or cultural incompatibility was not clear.

#### Learning together

Regardless of the difficulties encountered while providing health care to members of CALD communities, both focus group participants and providers believed their experiences were mutually beneficial, with many focus group participants often being satisfied with their care. The perception of racism or ill-treatment was not pervasive with Arabic, Chinese and Vietnamese participants reporting a high regard for Australian doctors, particularly those who took the time to treat them appropriately. When adequate information or treatment was provided, participants across the focus groups were satisfied:Interactions with patients from diverse backgrounds were reported by providers as being *“very positive and a huge learning curve”,* a *“rich experience”* and *“very rewarding”.*

"“The GP was patient with me and explained in depth and I was contented with the information”. (Sudanese)"

### Suggestions to improve health care provision for CALD communities

Both focus groups participants and healthcare providers believed improvements could be made in the delivery of health care services to members of the CALD community. In particular, these centred on improvements to human resourcing and increasing the provision of health-related information via various media and methods of dissemination (Table 6). These will be discussed in further detail below.

**Table 6:** Summary of suggestions for improvements to healthcare delivery by focus group participants and healthcare providers

**SUGGESTIONS FOR IMPROVEMENTS**	**Arabic**	**Sudanese**	**Tongan**	**Chinese**	**Vietnamese**	**Health-care Providers**
Increases in human resourcing						
More effective interpreters		x	x	x	x	x
More multicultural health professionals	x	x		x	x	x
More health providers in general	x	x		x	x	x
Increase in provision of health-related information						
General need for literacy	x	x	x	x	x	x
Chronic disease		x			x	x
Common disease in Australia		x			x	
Epidemics		x		x		
Prevention of back pain				x		
Mental illness		x				
Arthritis		x				
Healthy lifestyle		x				x
Alternative therapy		x				
Prevention/causes of illness		x				x
Health system		x		x		x
Preferred medium of health-related information						
Brochures	x	x				
TV/audio	x	x		x		
Radio			x	x	x	
Newspapers				x	x	
Internet			x	x		x
Preferred method of dissemination of health-related information						
Community associations/churches	x	x	x	x	x	x
GP or other health provider	x	x		x	x	x
Government via community association or directly	x	x	x	x		x

#### Improved interpreting services

Even though there was a general feeling that access to interpreter services had improved over time, many providers and focus groups participants still felt that such services should be expanded to provide a more comprehensive service (especially after-hours and for the elderly), that is gender-sensitive and extended to the relatives of the patient.

#### Increasing the cultural sensitivity of the health care system

Increasing cultural competence across the health care system was cited as essential. Many providers and focus group participants felt that health care provision would be facilitated if training in cultural awareness was made available or increased, in particular regarding patients from regions of political and religious unrest.

A low level of representation of CALD populations within the workforce was seen as contributing to a lack of resources being directed at CALD communities. This lack of cultural diversity or appreciation of multiculturalism within the wider community was seen by both providers and focus group participants to underlie the problem of cultural incompetence:

"”Australians are very ethnocentric and they’re not very culturally competent, the ones in Queensland anyway.” (HP3)"

"“Yes, sometimes the misunderstanding comes from cultural difference.” (Chinese)"

In addition to training existing staff, recruiting health-care workers from CALD communities was perceived as essential to improving health care delivery:

"“We need to train staff, but we must make sure that within our workforce we have more CALD people, more culturally diverse, so we can train staff that can work with CALD patients, but if we do not have those people, we need to work with the people from CALD groups to work in the area, primary and secondary prevention and treatment of chronic disease.” (HP6)"

Consequently, it was believed that the government should *“drive a multicultural agenda”* to engender a *“genuine reflection of our population in our workforce”,* in an effort to help improve health care outcomes for CALD communities. This solution was also cited by focus group participants at the more prosaic level and was seen to not only make service provision more efficient:to more empowering for the patient:and also more likely to be an acceptable form of intervention for CALD patients:

"“…three doctors came for us but they couldn’t identify the problem until a Taiwanese doctor came. In only a few minutes he was clear about the problem.” (Chinese)"

"“The patient can have the confidence to communicate with GPs in regard to their conditions because someone who understands them is there and can help.” (Sudanese)"

"“Health promotion campaign developed by the Tongan community, more Tongan participate.” (Tongan)"

“Health awareness sessions or campaigns similar to those conducted by you can help us because you bring our attention to things we are not aware of” (Arabic, where the speaker is referring to health promotion sessions conducted by Arabic speaking health workers).

#### Improving health promotion and community services

Health care providers bemoaned the lack of culturally appropriate courses and resources in general, particularly given their utility in Anglo-communities. It was generally acknowledged that *“people view their illnesses through their cultural awareness*” and that simply providing translation of material directed at the mainstream population was inadequate. Focus group participants wanted more culturally tailored health-related information made available to them using a variety of media ranging from leaflets, newspapers, radio and audio/visual means.It was apparent that an increase in community-based education programs was essential:

"“…in terms of health, one of the better things would be to form community organisations that got people togetherrdquo; so “that (the) CALD community can actually change with the support of their family and community.” (HP4)"

In particular, *“that community group could have that chronic disease focus where people get together and do things together in a culturally safe sort of way.” (HP4)* and for CALD communities where family is a crucial reference point the need to: *“Encourage family based physical activity.” (Tongan)* reflecting the need for lifestyle management interventions to be accordingly tailored to cultural needs.

Participants suggested making information available through health care providers, community and church organizations. Government organizations should liaise with these communities in order to improve health promotion.

## Discussion

This study highlights the importance of attention to culture, language and health literacy to enable adequate delivery of health services to people from culturally and linguistically diverse communities. A striking finding is the level of concordance between the CALD community members and health care providers regarding their perception of the provision of health-related services. Both groups were generally positive about their interactions with one another, whilst being aware of discriminatory practices within the health care system. Of particular concern to both parties were the barriers presented by language difficulties, cultural differences, health beliefs, lack of culturally competent health providers, limited educational resources, health literacy and low socio-economic status.

Our study emphasises that developing cultural awareness needs to focus on factors other than language. Simply overcoming the language barrier is insufficient to resolve the problems presented by cultural incompetence on behalf of the provider. Cultural competence is critical to understanding a patient’s needs and attitudes to health-related issues [16]. This includes appreciating dietary differences, perception of health and disease management [17] and is especially important in the context of treating populations where religious or strong cultural practices influence behaviour, for example integrating the requirement to fast during Ramadan in treatment plans for Type 2 diabetes mellitus in Muslims. Thus lack of culturally tailored information significantly impairs the ability of the CALD populations to make an informed decision about their health [18]. Participants of this study mention that should this approach be adopted, comprehension of disease and adherence to treatment plans would improve. Participants of this study indicated that when a culturally competent or tailored approach is adopted (which they describe for example as having access to health providers from their own cultural community or supporting grass roots community organisations) comprehension of disease and management and their willingness to engage in health-promotional activities increases.

Adopting a multicultural agenda across all levels of the health care system is also reported as essential in improving outcomes for CALD patients in Queensland’s strategic health plan [19]. Developing overall cultural and linguistic sensitivity requires change at the system, institutional, professional and individual provider levels, which encompasses shared responsibility by consumer and community engagement, gathering information, refining appropriate methodologies, evaluating the projects and sharing the findings. This recommendation reflects proposals outlined in the Australian National Health and Medical Research Council (NHMRC) Guide on Cultural Competence [20].

The role the community plays in providing support, particularly in the elderly, was also highlighted. The existing evidence indicates that some elderly people from non-English speaking backgrounds may present later than average to specialised services like memory clinics, although there is a bilingual specialist available [21]. Health providers emphasised the need to exploit existing or established community support networks that have a focus on health for CALD populations. Community support groups would provide a culturally sensitive arena from which patients could garner emotional support and access health-related information [22]. The success of community support is evident in the treatment of Type 2 diabetes mellitus in migrant populations [23] for example, and also depression in East African communities [24].

Participants in our study very clearly state that increasing CALD representation within the health workforce should not just be limited to increasing the number of interpreters, but extend to all health care professions [20]. Focus group participants and health care providers have had experiences with multicultural health workers and believe that increasing their availability would mitigate many of the problems both providers and patients face during cross-cultural interactions. Multicultural Health Workers (MHW) are trusted members of the CALD community who are trained to act as front line liaisons with their communities. The shared cultural and linguistic background creates mutual understanding between the patient and the worker which enables them to facilitate access to health services and to deliver culturally competent and quality health services to their community. The model is similar to that of Aboriginal Health Workers which has been developed to improve health care delivery for Indigenous Australians [25]. Perhaps lessons learned from the Indigenous health model can serve as a guideline for improving service delivery to CALD communities.

Health literacy was perceived as an important issue. Focus group participants found medical terminology particularly problematic despite some demonstrating high levels of English-proficiency. This has also been raised elsewhere [26]. Furthermore, translating medical terminology into simple language is particularly challenging if there is no language or conceptual equivalent of certain medical conditions in other cultures, for instance, when treating East African refugees for mental illness as the there is no word for ‘depression’ in Sudanese or Ethiopian [24]. These examples highlight not only the importance of limiting the use of medical terminology when treating CALD patients, but also recognising that Western medical concepts may not readily translate across cultures [26]. Educational resources are also needed to help develop health literacy in CALD populations. Improvements are not just required in the provision of services *per se*, but should also be geared towards amending the low levels health literacy of CALD populations [16,22,27].

Furthermore, taking these factors into consideration, we are ensuring that health inequities and inequalities, and social constructs of illness are addressed within preventative and primary health care, particularly in lifestyle related chronic conditions for CALD populations [16,17].

Although, this paper has highlighted some of the unmet health care needs for particular CALD communities they are important to understand the issues around delivery of the comprehensive health care services to multicultural society through monoculturally designed health care system [28].

### Limitations and strengths of the study

The main limitation of this study was that we used convenience sampling thereby possibly introducing bias according to Bryman [29]. Our focus group sample was limited to self-referred community members and who reside in the urban area of south east Queensland. We were careful to interview a range of health professionals who had varied experience servicing CALD communities. Nevertheless our sampling technique raises the possibility that not all concerns and opinions regarding health service delivery to members of the CALD population in this setting were uncovered by this study and that the accounts may be biased. The broad range of both positive and negative views and perceived issues raised are consistent, however, with other findings reported in the literature. The other limitation of the study was the lack of no comparisons with mainstream English speaking background population. This will be addressed in a future study. The strength of this research is that focus groups were conducted in the native languages and allowed community members at any level of English language proficiency to participate and express their views. It has minimised loss of data that often happens while using interpreters.

Most importantly this study examined the perceptions of both sides of the health care equation namely the patient and health practitioner. The striking degree in concordance between both parties regarding the provision of healthcare to CALD populations lends further weight to the assertion that improvements need to be made in providing care to these communities.

## Conclusion

Our study highlights the need for a different approach to cultural competence of health care services across all levels of the health system in order to improve health outcomes for members of CALD communities [30]. Linguistic and cultural incompatibilities between patient and health care provider need to be redressed in addition to providing resources for patients to understand health-related issues and manage their health [20]. Cultural competencies include recognising the multiple diversities within diversity which supports developing culturally tailored interventions and results in recognising all migrants and refugees being different in terms of health status and health care experiences, cultural and social and environmental determinants of health. Providing a culturally safe and sensitive environment and relevant multicultural workforce for patients to prevent, detect early or self-manage their chronic disease by establishing appropriate community support is also recommended.

## Competing interests

The author(s) declare that they have no competing interests.

## Authors' contributions

NK conceived the project and facilitated funding from ECCQ. NK supervised the focus group facilitators. MVD obtained funding through the Primary Health Care Research Evaluation and Development Program at Bond University (PHCRED) and facilitated training of focus group facilitators. SB analysed the data with NK. SB wrote the first draft, edited subsequent drafts and all authors contributed to the manuscript. MVD supervised and guided the project. All authors read and approved the final manuscript.

## Authors' information

NK was employed by ECCQ as chronic disease program manager at the time of the data collection. She is currently supported by a PHCRED novice research fellowship. SB is a post-doctoral researcher at the Discipline of General Practice, School of Medicine, The University of Queensland. She is also a visiting fellow at the Australian National University. MVD is Professor, Head of Discipline of General Practice, School of Medicine, The University of Queensland and visiting professor at Ghent University, Belgium. She was Professor of General Practice and Associate Dean of Research, Faculty of Health Sciences and Medicine, Bond University, Queensland, Australia at the time of data collection.

## Pre-publication history

The pre-publication history for this paper can be accessed here:

http://www.biomedcentral.com/1472-6963/12/322/prepub
